# Supplementary material for: Effects of sound exposure on the growth and intracellular macromolecular synthesis of E. coli k-12
Source: PeerJ. 2016 Apr 7;4:e1920. doi: 10.7717/peerj.1920 (PMC4830253; doi:10.7717/peerj.1920)
Supplement: Data S1 [file peerj-04-1920-s001.doc]

The data of the biomass of *E.coli* K-12 exposed to different sound frequency

| Treatment | Biomass value | | |
| --- | --- | --- | --- |
|  | Ⅰ | Ⅱ | Ⅲ |
| 250Hz | 1.111 | 1.268 | 1.143 |
| 500Hz | 1.181 | 1.194 | 1.225 |
| 1KHz | 1.170 | 1.197 | 1.302 |
| 2KHz | 1.291 | 1.294 | 1.333 |
| 4KHz | 1.266 | 1.247 | 1.219 |
| 6KHz | 1.159 | 1.137 | 1.037 |
| 8KHz | 1.360 | 1.317 | 1.436 |
| 10KHz | 1.191 | 1.171 | 1.097 |
| 12KHz | 1.090 | 1.169 | 1.116 |
| 14KHz | 1.083 | 1.173 | 1.155 |
| 16KHz | 1.186 | 1.167 | 1.229 |
| Control | 1.126 | 1.026 | 1.085 |

Note: The biomass of *E. coli* K-12 was represented by the maximum optical density at 600 nm. Ⅰ, Ⅱ, and Ⅲ represented three independent experiments under the same condition.

The data of the maximum specific growth rate (µ_max_) of *E.coli* K-12 exposed to different sound frequency

| Treatment | µ_max_ value (h^-1^) | | |
| --- | --- | --- | --- |
|  | Ⅰ | Ⅱ | Ⅲ |
| 250Hz | 1.658 | 1.619 | 1.757 |
| 500Hz | 1.738 | 1.625 | 1.914 |
| 1KHz | 1.795 | 1.590 | 1.664 |
| 2KHz | 1.829 | 1.990 | 2.034 |
| 4KHz | 1.782 | 1.751 | 1.555 |
| 6KHz | 1.762 | 1.841 | 1.671 |
| 8KHz | 1.946 | 1.934 | 2.003 |
| 10KHz | 1.813 | 1.844 | 1.929 |
| 12KHz | 1.803 | 1.827 | 1.695 |
| 14KHz | 1.799 | 1.807 | 1.752 |
| 16KHz | 1.776 | 1.803 | 1.608 |
| Control | 1.626 | 1.524 | 1.536 |

Note:Ⅰ, Ⅱ, and Ⅲ represented three independent experiments under the same condition.

The data of the biomass of *E.coli* K-12 exposed to different sound intensity level

| Treatment | Biomass value | | |
| --- | --- | --- | --- |
|  | Ⅰ | Ⅱ | Ⅲ |
| 70dB | 1.148 | 1.262 | 1.307 |
| 80dB | 1.352 | 1.323 | 1.438 |
| 90dB | 1.352 | 1.317 | 1.243 |
| 100dB | 1.220 | 1.281 | 1.198 |
| Control | 1.126 | 1.026 | 1.085 |

Note: The biomass of *E. coli* K-12 was represented by the maximum optical density at 600 nm. Ⅰ, Ⅱ, and Ⅲ represented three independent experiments under the same condition.

The data of the maximum specific growth rate (µ_max_) of *E.coli* K-12 exposed to different sound intensity level.

| Treatment | µ_max_ value (h^-1^) | | |
| --- | --- | --- | --- |
|  | Ⅰ | Ⅱ | Ⅲ |
| 70dB | 1.750 | 1.782 | 1.835 |
| 80dB | 1.951 | 1.918 | 2.014 |
| 90dB | 1.933 | 2.022 | 2.093 |
| 100dB | 2.023 | 2.159 | 2.271 |
| Control | 1.626 | 1.524 | 1.536 |

Note:Ⅰ, Ⅱ, and Ⅲ represented three independent experiments under the same condition.

The data of the biomass of *E.coli* K-12 exposed to different sound power level

| Treatment | Biomass value | | |
| --- | --- | --- | --- |
|  | Ⅰ | Ⅱ | Ⅲ |
| 55dB | 1.321 | 1.354 | 1.438 |
| 57dB | 1.651 | 1.534 | 1.648 |
| 59dB | 1.726 | 1.834 | 1.903 |
| 61dB | 1.801 | 1.843 | 1.945 |
| 63dB | 1.298 | 1.306 | 1.374 |
| Control | 1.126 | 1.026 | 1.085 |

Note: The biomass of *E. coli* K-12 was represented by the maximum optical density at 600 nm. Ⅰ, Ⅱ, and Ⅲ represented three independent experiments under the same condition.

The data of the maximum specific growth rate (µ_max_) of *E.coli* K-12 exposed to different sound power level

| Treatment | µ_max_ value (h^-1^) | | |
| --- | --- | --- | --- |
|  | Ⅰ | Ⅱ | Ⅲ |
| 55dB | 1.835 | 1.956 | 2.092 |
| 57dB | 3.576 | 3.444 | 2.943 |
| 59dB | 3.797 | 3.385 | 3.744 |
| 61dB | 3.977 | 3.548 | 3.986 |
| 63dB | 3.458 | 3.076 | 3.297 |
| Control | 1.626 | 1.524 | 1.536 |

Note:Ⅰ, Ⅱ, and Ⅲ represented three independent experiments under the same condition.

The data of the total intracellular protein in *E.coli* K-12 exposed to sound wave at different time

| Groups | Culture time (h) | The total intracellular protein (mg/g) | | | |
| --- | --- | --- | --- | --- | --- |
|  |  | Ⅰ | Ⅱ | Ⅲ |  |
| Control | 6  12  24  36  48 | 509.2  409.2  399.7  349.4  302.5 | 520  422.2  415.5  354.3  302.6 | 504.1  408.8  390.8  336.1  327.3 |  |
| Treatment | 6  12  24  36  48 | 574.3  459.0  399.7  349.9  316.7 | 565.1  470.4  413.2  352.2  304.2 | 559.8  452.4  397.9  371.9  326.2 |  |

Note:Ⅰ, Ⅱ, and Ⅲ represented three independent experiments under the same condition.

The data of the total intracellular RNA in *E.coli* K-12 exposed to sound wave at different time

| Groups | Culture time (h) | Total intracellular RNA (mg/g) | | | |
| --- | --- | --- | --- | --- | --- |
|  |  | Ⅰ | Ⅱ | Ⅲ |  |
| Control | 6  12  24  36  48 | 93.5  68.3  63.9  46.9  31.4 | 87.3  63.5  59.8  44.6  30.8 | 89.5  67.1  65.3  39.6  32.9 |  |
| Treatment | 6  12  24  36  48 | 110.2  70.6  63.1  48.9  36.3 | 110.8  69.8  61.3  45.7  36.2 | 118.0  77.4  66.4  49.1  32.5 |  |

Note:Ⅰ, Ⅱ, and Ⅲ represented three independent experiments under the same condition.

The data of the length of *E.coli* K-12 cells exposed to sound wave at 48 h

| Groups Number length of *E.coli* K-12 cells  of cells |
| --- |
| Control 100 1.84 1.84 2.50 2.24 1.58 2.11 1.58 1.32 1.71 2.50 1.58 1.58 1.71 1.97 1.84 1.45 1.58 2.63 2.24 1.84 2.16 2.15 2.07 1.46 1.59 1.47 1.54 2.11 1.59 1.94 2.38 2.26 1.58 1.32 1.84 1.92 1.57 1.62 1.78 1.94 1.81 2.50 2.24 1.58 1.84 1.36 1.09 1.05 2.01 1.68 1.71 2.50 1.85 1.78 1.32 1.97 1.69 1.65 2.26 2.03 2.56 2.58 1.59 2.35 2.33 1.92 2.59 1.89 1.85 1.67 1.66 2.06 1.71 2.14 2.08 2.34 2.18 1.49 1.33 2.25 1.49 1.74 2.36 2.42 1.80 2.30 2.16 1.22 2.50 1.72 2.41 1.68 1.88 1.82 2.09 2.06 1.83 1.40 1.77 1.12  TreatmentⅠ 100 1.18 2.90 1.58 1.71 2.50 2.24 2.63 2.37 1.84 1.97 2.50 1.58 1.97 2.11 1.58 1.58 2.37 2.37 2.76 1.45 2.55 1.62 1.67 1.17 2.51 2.31 1.63 2.56 2.37 2.57 1.67 1.62 1.76 1.87 2.36 1.94 1.91 2.41 1.99 1.44 2.43 1.09 1.38 1.94 1.68 1.65 1.98 1.97 1.76 1.91 1.88 1.77 1.89 2.21 1.68 1.59 2.42 2.41 2.46 1.65 2.44 1.34 1.56 1.89 1.55 2.67 2.15 2.34 1.54 2.03 2.38 1.64 1.77 2.04 1.64 2.45 2.22 2.34 2.44 2.05 1.85 1.79 2.02 3.18 1.62 2.00 2.33 3.75 3.84 1.96 1.97 2.88 2.39 1.55 1.82 2.41 2.20 2.34 2.33 2.45  TreatmentⅡ 100 1.97 1.58 5.26 2.37 2.90 2.76 2.63 1.32 1.58 3.03 2.50 1.45 1.45 3.03 2.50 1.58 2.37 3.29 2.11 2.241.39 2.40 2.37 4.28 3.15 1.12 3.03 4.02 3.89 3.67 1.36 1.83 1.85 1.51 3.09 2.11 2.54 2.83 1.26 1.18 2.84 3.50 1.34 1.46 1.29 2.43 2.01 3.04 1.57 3.09 1.85 2.37 3.48 1.51 2.54 3.12 1.41 1.59 3.40 1.65 2.68 1.96 2.55 1.86 1.77 2.56 3.10 3.49 2.45 2.67 1.42 2.34 2.92 1.26 3.43 1.39 4.02 1.36 3.04 3.09 1.40 1.89 1.58 5.13 2.46 2.67 3.16 3.52 2.46 2.01 2.81 1.56 2.45 1.30 1.87 1.80 4.65 1.25 2.42 1.46 |

Note: TreatmentⅠ : Cells exposed to sound intensity 80 dB, TreatmentⅡ : Cells exposed to sound intensity 100 dB.
