# Supplementary figures and images for: Effects of sound exposure on the growth and intracellular macromolecular synthesis of E. coli k-12
Source: PeerJ. 2016 Apr 7;4:e1920. doi: 10.7717/peerj.1920 (PMC4830253; doi:10.7717/peerj.1920)

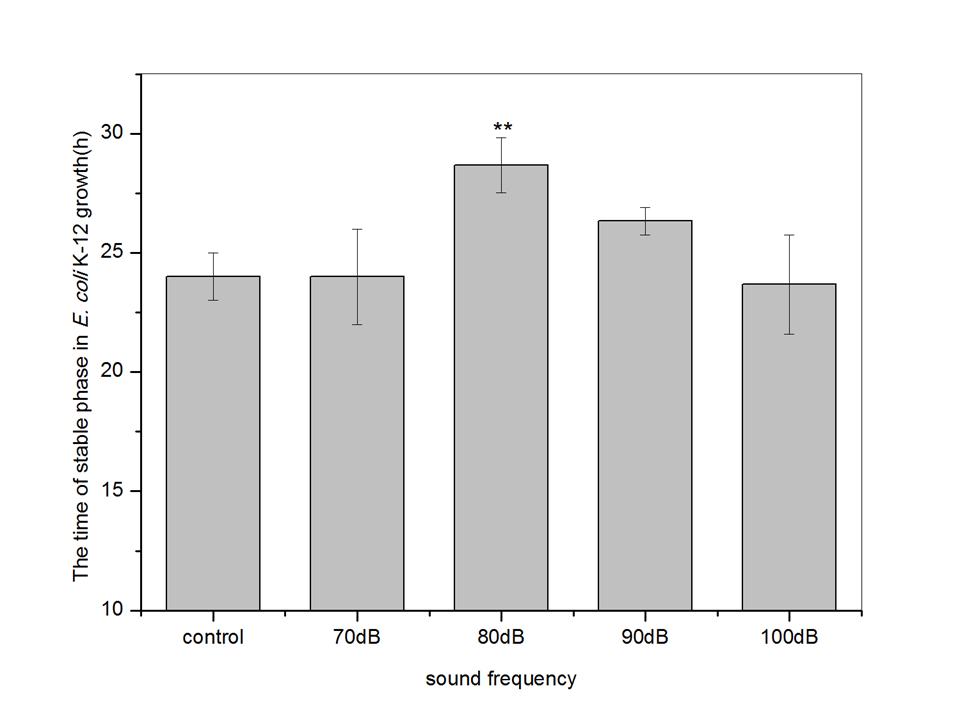

Supplement: Figure S1 — All experiments were stimulated at sound frequency 8 KHz and power 55 dB. Asterisks indicate significance: ∗∗0.001 < p < 0.01. Vertical bars represent means ±SD. [file peerj-04-1920-s002.png]
